# Improvement of Damage in Human Dermal Fibroblasts by 3,5,7-Trimethoxyflavone from Black Ginger (*Kaempferia parviflora*)

**DOI:** 10.3390/antiox11020425

**Published:** 2022-02-19

**Authors:** Sullim Lee, Taesu Jang, Ki Hyun Kim, Ki Sung Kang

**Affiliations:** 1 Department of Life Science, College of Bio-Nano Technology, Gachon University, Seongnam 13120, Korea; sullimlee@gachon.ac.kr; 2 College of Medicine, Dankook University, Cheonan 31116, Korea; jangts@dankook.ac.kr; 3 School of Pharmacy, Sungkyunkwan University, Suwon 16419, Korea; 4 College of Korean Medicine, Gachon University, Seongnam 13120, Korea

**Keywords:** human dermal fibroblasts, tumor necrosis factor-α, *Kaempferia parviflora*, 3,5,7-trimethoxyflavone, improvement of skin damage

## Abstract

Reactive oxygen species (ROS) are generated during intrinsic (chronological aging) and extrinsic (photoaging) skin aging. Therefore, antioxidants that inhibit ROS production may be involved in delaying skin aging. In this study, we investigated the potential effects of compounds isolated from black ginger, *Kaempferia parviflora*, a traditional medicinal plant, on normal human dermal fibroblasts in the context of inflammation and oxidative stress. The isolated compounds were structurally characterized as 5-hydroxy-7-methoxyflavone (**1**), 3,7-dimethoxy-5-hydroxyflavone (**2**), 5-hydroxy-3,7,3,4-tetramethoxyflavone (**3**), 7,4-dimethylapigenin (**4**), 3,7,4-trimethylkaempferol (**5**), and 3,5,7-trimethoxyflavone (**6**), using nuclear magnetic resonance spectroscopy (NMR) and liquid chromatography–mass spectrometry (LC/MS) analyses. These flavonoids were first evaluated for their ability to suppress extracellular matrix degradation in normal human dermal fibroblasts. Of these, 3,5,7-trimethoxyflavone (**6**) significantly inhibited the tumor necrosis factor (TNF)-α-induced high expression and secretion of matrix metalloproteinase (MMP)-1 by cells. We further found that 3,5,7-trimethoxyflavone suppressed the excessive increase in ROS, mitogen-activated protein kinases (MAPKs), Akt, and cyclooxygenase-2 (COX-2)and increased heme oxygenase (HO)-1 expression. The expression of pro-inflammatory cytokines, including interleukin (IL)-1β, IL-6, and IL-8, was also suppressed by 3,5,7-trimethoxyflavone (6). Taken together, our results indicate that 3,5,7-trimethoxyflavone (**6**) isolated from *K. parviflora* is a potential candidate for ameliorating skin damage.

## 1. Introduction

The skin is a major defensive organ formed from the human ectoderm; it performs sensory, control, and protective functions when in direct contact with latent harmful factors [1]. Intrinsic aging appears with a gradual decrease in cell activity of skin with age, and is caused by reactive oxygen species (ROS) produced from the cell metabolic process [2,3]. External aging and skin damage occur upon direct exposure to the external environment and are caused by environmental hazards, such as pollution, chemicals, smoking, and ultraviolet (UV) radiation [4,5]. UV radiation substantially contributes to various skin injuries and diseases, including skin aging and inflammatory skin diseases [6,7]. UV radiation induces intracellular ROS formation, causing widespread inflammatory damage from the epidermis to the dermis, resulting in accumulative skin damage, such as skin pigmentation and photoaging [8,9]. ROS generation causes intracellular oxidative damage and changes, such as DNA denaturation, cell membrane destruction, and inflammatory responses, causing functional problems [10,11,12]. Harmful alien substances can cause accumulation of ROS, which are produced by oxidative phosphorylation in the mitochondria [13]. Excessive ROS in the skin induces wrinkle formation because of cleavage of elastic fibers and fibrous proteins, such as collagen, which comprise the skin extracellular matrix (ECM) [6]. Matrix metalloproteinase-1 (MMP-1) is a collagenase that degrades collagen [14]. The major cause of extrinsic aging is a complex reaction induced by ROS, which is produced by the absorption of ultraviolet light by the skin. [15]. Therefore, substances that inhibit ROS and MMP-1 production can be effective in preventing skin damage, such as skin aging. 

When the skin is exposed to UV light, inducible nitric oxide synthase (iNOS), cyclooxygenase-2 (COX-2), tumor necrosis factor-α (TNF-α), and interleukins (ILs) are directly or indirectly induced [16,17,18]. In particular, skin fibroblast damage caused by the inflammatory responses accelerates photoaging [16,19]. The hypersecretion of TNF-α increased by UV B stimulates immune cells to promote the production of collagenase, which leads to ECM breakdown and skin aging such as wrinkles. Suppression of collagen production and induction of collagen degradation results in negative changes, such as loss of elasticity and wrinkles [20]. Further, long-term exposure to ultraviolet rays makes inflammation chronic, which causes diverse skin diseases. Therefore, many previous studies have examined skin wrinkle improvement, focusing on collagenase inhibition [21,22,23].

The inflammatory cytokine TNF-α, secreted by keratinocytes and UV irradiated fibroblasts, plays an important role in mediating skin aging as well as inflammatory diseases. With respect to skin aging, TNF-α suppresses collagen biosynthesis in skin fibroblasts and regulates collagen production through various signaling pathways [24]. Further, TNF-α induces the production of MMP interstitial collagenase (MMP-1) and the breakdown of collagen fibers [24,25]. Therefore, TNF-α activity regulation can be a powerful approach for developing new treatments for preventing inflammation related skin diseases and aging.

*K. parviflora*, also known as Thai ginseng or black ginger, is a medicinal plant belonging to the family Zingiberaceae. *K. parviflora*, known as Krachaidum in Thailand, is native to northern and northeastern Thailand. Its rhizomes are popular as a commercial health-promoting herb, and it has been used in traditional medicine to treat various diseases, including ulcers, inflammation, gout, allergy, abscesses, and osteoarthritis [26,27]. A number of previous pharmacological studies on the rhizome of *K. parviflora* have revealed its valuable therapeutic effects, including antimicrobial, anti-inflammatory, antiallergic, antioxidative, sex-enhancing, neuroprotective, vascular relaxation, anticancer, and cardioprotective activities [28]. Due to the pharmacological properties of its rhizomes, *K. parviflora* has been thoroughly investigated for its bioactive phytochemicals [29,30], and methoxyflavones have been identified as the primary bioactive constituents in *K. parviflora*. A representative compound in *K. parviflora* rhizomes, 5,7,3′,4′-tetramethoxyflavone, exhibits diverse pharmacological properties [31,32]. The β-catenin and EP/cAMP/PKA signaling pathways were significantly inhibited by 5,7,3′,4′-tetramethoxyflavone in cultured rat chondrocytes, leading to articular cartilage and chondrocyte protection [31]. In addition, 5,7,3′,4′-tetramethoxyflavone exerted antiplasmodial effects against *Plasmodium falciparum*, with IC_50_ values of 3.70 μg/mL [32]. In this context, diverse bioactive compounds from *K. parviflora* have been attractive to natural product chemists for the discovery of novel bioactive agents.

As part of a continuing study for discovering bioactive natural products from natural resources [33,34,35,36], we explored potential bioactive flavonoids from *K. parviflora*, a traditional medicinal plant, because the antioxidant activity of such flavonoids can suppress intracellular oxidative damage and prevent oxidative stress-related damage to the skin ECM [37,38]. In this study, we separated methoxyflavones from a methanol extract of *K. parviflora* rhizomes using column chromatography and high-performance liquid chromatography (HPLC) under the guidance of liquid chromatography-mass spectrometry (LC-MS)-based analysis. We further examined their protective effects against TNF-α-induced aging damage to normal human dermal fibroblasts (NHDFs) and assessed the mechanism of action of these compounds.

## 2. Materials and Methods

### 2.1. Plant Material

*K. parviflora* rhizomes were collected in January 2020 from Chiang Mai City, northern Thailand. This material was authenticated by K. H. Kim. A voucher specimen (SKKU-BG 1908) was stored in the herbarium of the School of Pharmacy, Sungkyunkwan University, Suwon, Korea. 

### 2.2. Extraction of K. parviflora Rhizomes and Separation/Isolation of Compounds

Dried *K. parviflora* rhizomes (132 g) were crushed and then extracted with 80% MeOH/H_2_O (2.0 L, 24 h × 2) at room temperature, and then the residue of *K. parviflora* rhizomes were extracted with 80% MeOH/H_2_O (3.0 L, 12 h) under reflux. The filtered extracts were extracted at 20 ± 5 °C, were combined under reflux, and then evaporated under reduced pressure to obtain a crude MeOH extract (9.2 g) using a rotary evaporator. The resultant extract was applied to suspension using 700 mL of distilled water, and the suspended extract was solvent-partitioned by employing four organic solvents (each 700 mL), specifically hexane, dichloromethane (CH_2_Cl_2_), ethyl acetate (EtOAc), and *n*-butanol (BuOH), three times. As a result, four fractions, the hexane-soluble (1.0 g), CH_2_Cl_2_-soluble (3.2 g), EtOAc-soluble (0.4 g), and *n*-BuOH-soluble (0.5 g) fractions were obtained. LC-MS analysis of each fraction was carried out on an Agilent 1200 Series HPLC equipment (Agilent Technologies, Santa Clara, CA, USA), furnished with a diode array detector (DAD) and 6130 Series ESI mass spectrometer, using an analytical Kinetex C18 100 Å column (Phenomenex, Torrance, CA, USA, 100 × 2.1 mm, 5 μm) at flow rate of 0.3 mL/min. Based on the reference to a house-built UV library database, LC-MS analysis of the four fractions revealed the presence of the majority of flavonoids in the hexane-soluble fraction. Thin-layer chromatography was performed to detect the spots of compounds using precoated silica gel F_254_ plates and RP-C_18_ F_254s_ plates (Merck, Darmstadt, Germany). The hexane-soluble fraction (1.0 g) was subjected to silica gel column chromatography (eluted with *n*-hexane/EtOAc [10:1→1:1], CH_2_Cl_2_/MeOH [10:1→1:1], gradient solvent system) to obtain six fractions (H1–H6). Fraction H2 (91.5 mg) was separated by Sephadex LH-20 column chromatography (CH_2_Cl_2_/MeOH 2:8]) to obtain five subfractions (H21–H25). Subfraction H22 (29.2 mg) was isolated using semi-preparative reverse-phase HPLC with 78% MeOH/H_2_O (flow rate of 2 mL/min) to obtain compounds **1** (1.5 mg) and **2** (2.4 mg). Fraction H5 (112.7 mg) was subjected to Sephadex LH-20 column chromatography [CH_2_Cl_2_/MeOH (2:8)] to obtain two subfractions (H51 and H52). Subfraction H52 (28.2 mg) was separated using semi-preparative reverse-phase HPLC with 83% MeOH/H_2_O (flow rate of 2 mL/min) to isolate compounds **3** (2.2 mg), **4** (2.5 mg), and **5** (0.5 mg). Finally, fraction H6 (271.8 mg) was fractionated by Sephadex LH-20 column chromatography (CH_2_Cl_2_/MeOH [2:8]) to obtain four subfractions (H61–H64). Subfraction H64 (46.3 mg) was isolated using semi-preparative reverse-phase HPLC with 78% MeOH/H_2_O (flow rate of 2 mL/min) to obtain compound **6** (3.0 mg). 

### 2.3. Cell Culture and Sample Preparations

We purchased NHDFs from PromoCell GmbH (Heidelberg, Germany). Cells were maintained in Dulbecco’s modified Eagle medium (Corning, Manassas, VA, USA). The medium consisted of 10% fetal bovine serum (Atlas, Fort Collins, CO, USA) and 100 U/mL penicillin-streptomycin solution (Gibco, Grand Island, NY, USA). Cells were cultured in a humid atmosphere containing 5% CO_2_ at 37 °C. The six isolated compounds for cell treatment were prepared by dissolving them in dimethyl sulfoxide (DMSO; Sigma-Aldrich, St. Louis, MO, USA), and the final concentration was kept below 0.1%. TNF-α (PeproTech, Rocky Hill, NJ, USA) was prepared by dissolving in 1% bovine serum albumin. 

### 2.4. Cell Viability

We seeded NHDFs at 1 × 10^4^ cells/well in 96-well plates and incubated them for 24 h. To create starvation conditions, the medium was then exchanged for serum-free. Serum starvation drives all cells to a phase of growth arrest, making all cells have the same cell cycle [39]. After 24 h, the NHDFs were exposed to each concentration of the compound. After 24 h, to measure cell viability, EZ-Cytox solution (Dogen, Seoul, Korea) was added to each cell-containing well, and absorbance was then determined after incubation for 2 h. The measurement was performed using a SPARK 10M device (Tecan Group Ltd., Männedorf, Switzerland), and the wavelength was set to 450/600 nm. Cell viability for each sample was presented as percent of the vehicle control. 

### 2.5. Evaluation of Intracellular ROS

We seeded NHDFs at 1 × 10^4^ cells/well in 96-well black plates, incubated them for 24 h, and then replaced the medium with serum-free medium to create starvation conditions. After 24 h, cells were treated with 3,5,7-trimethoxyflavone for 24 h. The cells were then exposed to 20 ng/mL TNF-α (PeproTech, Rocky Hill, NJ, USA) for 15 min. After 15 min of incubation, staining was carried out with dichlorofluorescein diacetate (DCFDA; Sigma-Aldrich) and washed with phosphate-buffered saline (Welgene, Gyeongsangbuk, Korea). Fluorescence imaging was performed using a fluorescence microscope IX51 (Olympus, Tokyo, Japan). Fluorescence was measured using a SPARK 10M, and the wavelength was set to 485/535 nm. Results of intracellular ROS were presented as percent of the vehicle control. 

### 2.6. Real-Time Quantitative Reverse Transcription PCR

We seeded NHDFs at 3 × 10^5^ cells/well in 6-well plates, incubated them for 24 h, and then replaced the medium with a serum-free medium to create starvation conditions. After 24 h, cells were treated with 3,5,7-trimethoxyflavone for 24 h. The cells were then exposed to 20 ng/mL TNF-α for 15 min. To measure expression of IL-1β, IL-6, IL-8, and β-actin, the cells were harvested after 4 h. To test for MMP-1 and procollagen I α1(COLIA1), the cells were harvested after 24 h. Next, the cells were harvested and isolated to cellular RNA by a RNeasy Mini Kit (QIAGEN, Hilden, Germany). Complementary DNA synthesis was performed using the RevertAid First Strand cDNA Synthesis Kit (Thermo Fisher Scientific, Waltham, MA, USA). Real-time PCR was carried out using PowerUp SYBR PCR Master Mix (Applied Biosystems, Waltham, MA, USA). The reaction was performed using the QuantStudio™ 3 Real-Time PCR System (Applied Biosystems), and the thermal conditions were set to 40 cycles (95 °C for 15 s, 60 °C for 30 s, 95 °C for 30 s). The primer sequences are shown in a previous paper [40], and β-actin was used as the reference gene.

### 2.7. Enzyme-Linked Immunosorbent Assay (ELISA)

We seeded NHDFs at 2 × 10^4^ cells/well in 48-well plates, incubated them for 24 h, and then replaced the medium with a serum-free medium to create starvation conditions. After 24 h, the NHDF was treated with 3,5,7-trimethoxyflavone for 1 h, followed by exposure to 20 ng/mL of TNF-α. To measure protein expression for IL-1β, IL-6, and IL-8, the medium was collected from cells after 12 h. To measure protein expression for MMP-1 and procollagen I α1 (COLIA1), the medium was collected from cells after 24 h. Protein secretion was determined using a human Total MMP-1 DuoSet ELISA kit and a Human Pro-Collagen I alpha 1 DuoSet ELISA (R&D systems, Minneapolis, MN, USA). The absorbance was measured using a SPARK 10M spectrophotometer, and the wavelength was set to 550/600 nm. Results of protein secretion were presented as percent of the vehicle control.

### 2.8. Western Blotting

We seeded NHDFs at 3 × 10^5^ cells/well in 6-well plates, incubated them for 24 h, and then replaced the medium with a serum-free medium to create starvation conditions. After 24 h, cells were treated with 3,5,7-trimethoxyflavone for 24 h. The cells were then exposed to 20 ng/mL TNF-α. To test for phospho-ERK, ERK, phospho-p38, p38, phospho-JNK, JNK, and GAPDH, the cells were collected after 15 min. To test for phospho-Akt, Akt, COX-2, heme oxygenase 1 (HO-1), and GAPDH, the cells were collected after 6 h. The cells were lysed with 1× radioimmunoprecipitation assay (RIPA) buffer (Tech & Innovation, Gangwon, Korea). The lysate was centrifuged, and the supernatant was prepared as eluted protein samples. The protein concentration was determined using a BCA Protein Assay Kit (Merck). Equal protein levels were analyzed using Western blotting. The primary antibodies, phospho-ERK, ERK, phospho-p38, p38, phospho-JNK, JNK, phospho-Akt, Akt, COX-2, HO-1, COX-2, and GAPDH (Cell Signaling Technology, Danvers, MA, USA) were reacted for 4 h at 20 ± 5 °C. The secondary antibodies, goat anti-rabbit IgG-HRP, and goat anti-mouse IgG-HRP (Santa Cruz Biotechnology, Dallas, TX, USA), were incubated for 1 h at room temperature. Protein bands were visualized using SuperSignal^®^ West Femto Maximum Sensitivity Chemiluminescent Substrate (Thermo Fisher Scientific) and Fusion Solo Chemiluminescence System (PEQLAB Biotechnologie GmbH, Erlangen, Germany). The band densities were calculated based on the ratio to the GAPDH band. Results were calculated from band density and were presented as percent of vehicle control. 

### 2.9. Statistical Analysis

The experimental data are presented as mean ± standard error of the mean (SEM). Differences of each group were assessed by using one-way analysis of variance (ANOVA) followed by Tukey’s HSD (honestly significant difference) test. A *p*-value of 0.05 or less was considered statistically significant.

## 3. Results

### 3.1. Isolation and Structural Identification of Compounds

**Figure 1:**
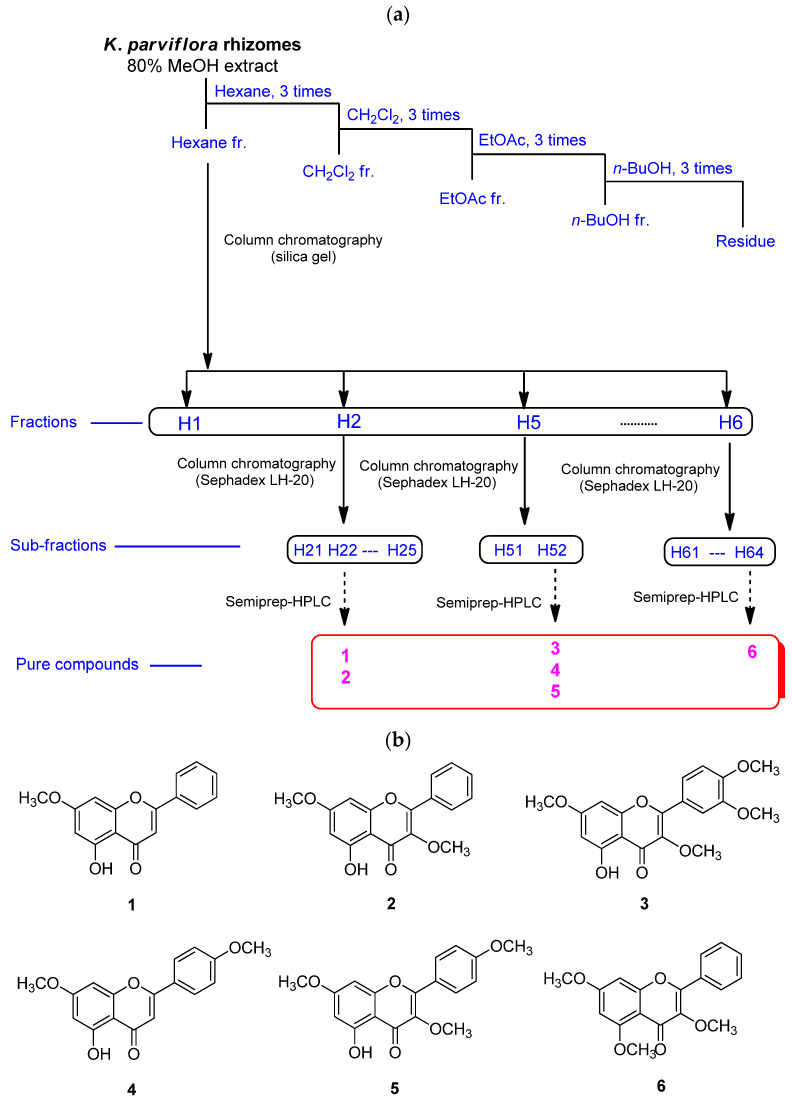
The separation scheme (**a**) and the chemical structures (**b**) of compounds **1**–**6**.

The MeOH extract of *K. parviflora* rhizomes was sequentially subjected to solvent partitioning by employing four organic solvents, specifically hexane, CH_2_Cl_2_, EtOAc, and *n*-BuOH, to yield four fractions. As a result of LC-MS analysis of each fraction, it was confirmed that the hexane fraction had the highest content of flavonoids with antioxidant potential. Six methoxyflavones (**1**–**6**; Figure 1) were isolated from the hexane fraction using repeated column chromatography and semi-preparative HPLC separation. These isolated methoxyflavones were structurally characterized as 5-hydroxy-7-methoxyflavone (**1**) [41], 3,7-dimethoxy-5-hydroxyflavone (**2**) [42], 5-hydroxy-3,7,3′,4′-tetramethoxyflavone (**3**) [43], 7,4′-dimethylapigenin (**4**) [41], 3,7,4′-trimethylkaempferol (**5**) [44], and 3,5,7-trimethoxyflavone (**6**) [45] by comparing their nuclear magnetic resonance spectroscopic data (Figures S1–S6) with those previously reported as well as MS data obtained from LC-MS analyses. 

### 3.2. Effect of Flavonoids 1–6 Isolated from K. parviflora on Viability of NHDFs

**Figure 2:**
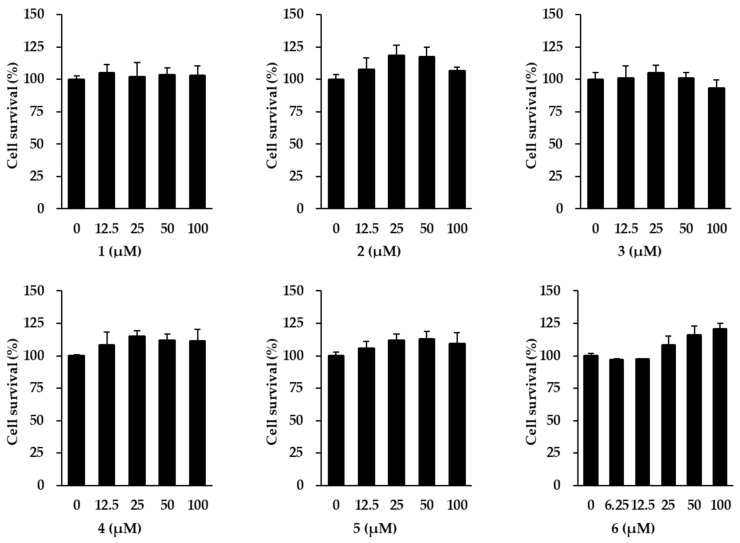
Survival of normal human dermal fibroblasts (NHDFs) under treatment with flavonoids 1–6 isolated from *K. parviflora*. We seeded NHDFs at 1 × 10^4^ cells/well in 96-well plates, incubated them for 24 h, and replaced the medium with a serum-free medium to create starvation conditions. After 24 h, NHDFs were exposed to each concentration of the compounds. After 24 h, the measurement of cell viability was conducted with EZ-Cytox solution. Results of cell viability were presented as percent of the vehicle control. The results were obtained through three replicate experiments, and the graphs are represented as mean ± SEM.

Ahead of investigating the anti-aging effects of flavonoids **1–6** isolated from *K. parviflora*, their effects on NHDF cell viability were measured. As shown in Figure 2, flavonoids **1–6** did not exhibit considerable cytotoxicity against NHDFs at 100 µM. Therefore, all flavonoids were used in subsequent experiments at concentrations up to 100 μM.

### 3.3. Effect of Flavonoids Isolated from K. parviflora on MMP-1 Secretion in TNF-α-Stimulated NHDFs

**Figure 3:**
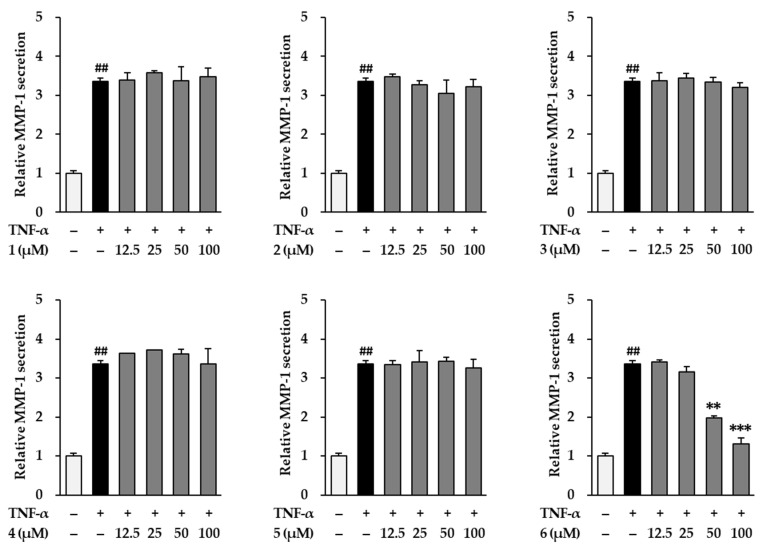
Effects of flavonoids 1–6 isolated from *K. parviflora* on MMP-1 secretion in normal human dermal fibroblasts (NHDFs). We seeded NHDFs at 2 × 10^4^ cells/well in 48-well plates, incubated them for 24 h, and replaced the medium with a serum-free medium to create starvation conditions. After 24 h, cells were treated with 50 and 100 μM of 6 for 1 h, and the cells were exposed to 20 ng/mL TNF-α for 24 h. After 24 h, we measured MMP-1 secretion with an ELISA kit. Results of MMP-1 secretion were presented as percent of the vehicle control. The results were obtained through three replicate experiments, and the graphs are represented as mean ± SEM. ^##^ *p* < 0.01 vs. vehicle control. ** *p* < 0.01 and *** *p* < 0.001 vs. TNF-α stimulated control.

We then investigated the inhibitory effects of the six flavonoids (1–6) by measuring the MMP-1 secretion by TNF-α-stimulated NHDFs. As shown in Figure 3, TNF-α (20 ng/mL) significantly increased MMP-1 secretion by 3.37 ± 0.08-fold (*p* < 0.01) compared with that of the vehicle control. Among the six flavonoids from *K. parviflora*, 3,5,7-trimethoxyflavone (**6**) potently reduced the secretion of MMP-1 induced by TNF-α. Treatment with 50 and 100 µM **6** suppressed the secretion of MMP-1 to 1.99 ± 0.05-fold (*p* < 0.01) and 1.76 ± 0.06-fold (*p* < 0. 01), compared with that of the control. Therefore, we focused on 3,5,7-trimethoxyflavone in further studies to elucidate the mechanism by which it demonstrates protective effects against NHDF damage by TNF-α.

### 3.4. Effect of 3,5,7-Trimethoxyflavone on Intracellular ROS Accumulation in TNF-α-Stimulated NHDFs

**Figure 4:**
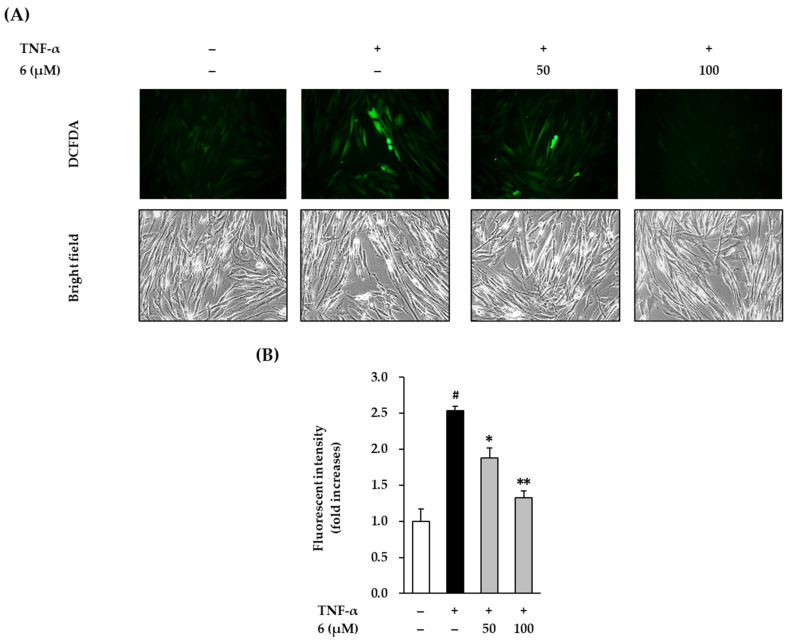
Effects of 3,5,7-trimethoxyflavone (6) on intracellular ROS accumulation in TNF-α stimulated normal human dermal fibroblasts (NHDFs). (**A**) Bands of protein expressions. (**B**) Bar graphs of the relative expression. We seeded NHDFs at 1 × 10^4^ cells/well in 96-well black plates incubated them for 24 h, and replaced the medium with a serum-free medium to create starvation conditions. After 24 h, cells were treated with 50 and 100 μM of 6 for 1h, and the cells were exposed to 20 ng/mL TNF-α for 15 min. The cell was stained with dichlorofluorescin diacetate (DCFDA) for 15 min, and photographs was observed with a microscope IX51. The measurement of fluorescence was conducted using a SPARK 10M. Results of intracellular ROS were presented as a percent of the vehicle control. The results were obtained through three replicate experiments, and the graphs are represented as mean ± SEM. ^#^ *p* < 0.05 vs. vehicle control. * *p* < 0.05 and ** *p* < 0.01 vs. TNF-α stimulated control.

TNF-α is a stimulator that can cause oxidative stress in NHDFs. Therefore, we evaluated the effect of 3,5,7-trimethoxyflavone on ROS accumulation in TNF-α-stimulated NHDFs. As shown in the Figure 4, exposure of 20 ng/mL TNF-α did show dramatically increased ROS accumulation by 2.54 ± 0.06-fold (*p* < 0.05) compared with that in the vehicle control. In contrast, 3,5,7-trimethoxyflavone (6) significantly reduced ROS accumulation. Treatment with 50 and 100 µM 6 suppressed ROS production to 1.88 ± 0.14-fold (*p* < 0.05) and 1.33 ± 0.09-fold (*p* < 0.01), respectively, compared with that in the control. 

### 3.5. Effect of 3,5,7-Trimethoxyflavone on TNF-α-Induced Phosphorylation of MAPKs in NHDFs

**Figure 5:**
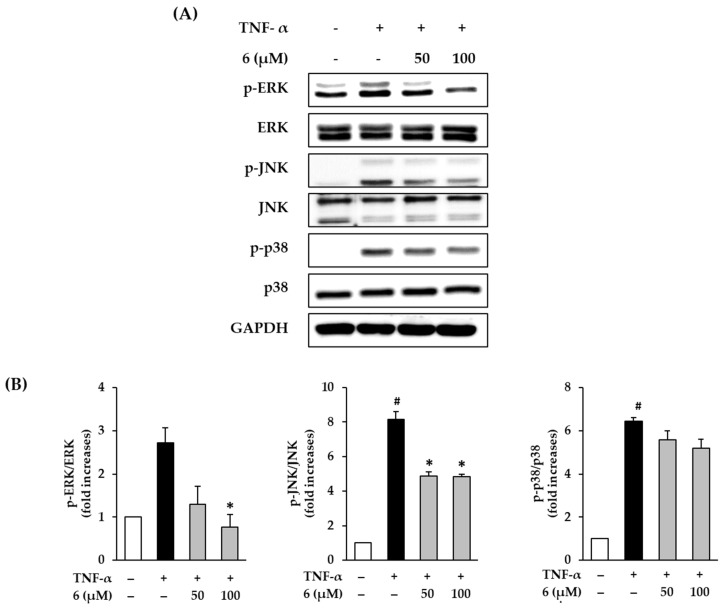
Effects of 3,5,7-trimethoxyflavone (6) on MAPK phosphorylation in normal human dermal fibroblasts (NHDFs) stimulated by TNF-α. (**A**) Bands of protein expressions. (**B**) Bar graphs of the relative expression. NHDFs were seeded at a density of 3 × 10^5^ cells/well in 6-well plates and incubated for 24 h, and then the medium was replaced with a serum-free medium to create starvation conditions. After 24 h, cells were treated with 50 and 100 μM of 6 for 1 h, and the cells were exposed to 20 ng/mL TNF-α for 15 min. Relative comparison of expression levels of p-ERK, ERK, p-JNK, JNK, p-p38, p38, and GAPDH proteins were determined with Western blotting. Results of each protein expression was presented as percent of the vehicle control. The results were obtained through three replicate experiments, and the graphs are represented as mean ± SEM. ^#^ *p* < 0.05 vs. vehicle control. * *p* < 0.05 vs. TNF-α stimulated control.

To improve the understanding of the mechanism of the cytoprotective action of 3,5,7-trimethoxyflavone (**6**), we evaluated its effect on MAPK phosphorylation in TNF-α-stimulated NHDFs. MAPK protein expression was analyzed using Western blotting. As shown in Figure 5, exposure of 20 ng/mL TNF-α did show activation by of ERK, JNK, and p38 in NHDFs, which was reduced upon treatment with 6. ERK phosphorylation by TNF-α-stimulation was shown to increase by 2.72 ± 0.35-fold, whereas treatment with **6** suppressed ERK phosphorylation to 1.30 ± 0.42-fold (50 µM) and 0.77 ± 0.30-fold (100 µM, *p* < 0.05) compared with that in the control. JNK phosphorylation by TNF-α-stimulation was shown to increase by 8.15 ± 0.45-fold, whereas treatment with **6** suppressed it by 4.88 ± 0.24-fold (50 µM, *p* < 0.05), and 4.86 ± 0.13-fold (100 µM, *p* < 0.05) of the control. Further, TNF-α stimulation increased p38 phosphorylation by 6.43 ± 0.17-fold, whereas treatment with **6** suppressed it by 5.58 ± 0.42-fold (50 µM), and 5.20 ± 0.31-fold (100 µM) compared with the control; however, this difference was not significant. These results indicate that 3,5,7-trimethoxyflavone (**6**) can suppress the MAPK phosphorylation induced by TNF-α stimulation.

### 3.6. Effect of 3,5,7-Trimethoxyflavone on Akt Phosphorylation and Expression of COX-2 and HO-1 in TNF-α-Stimulated NHDFs

**Figure 6:**
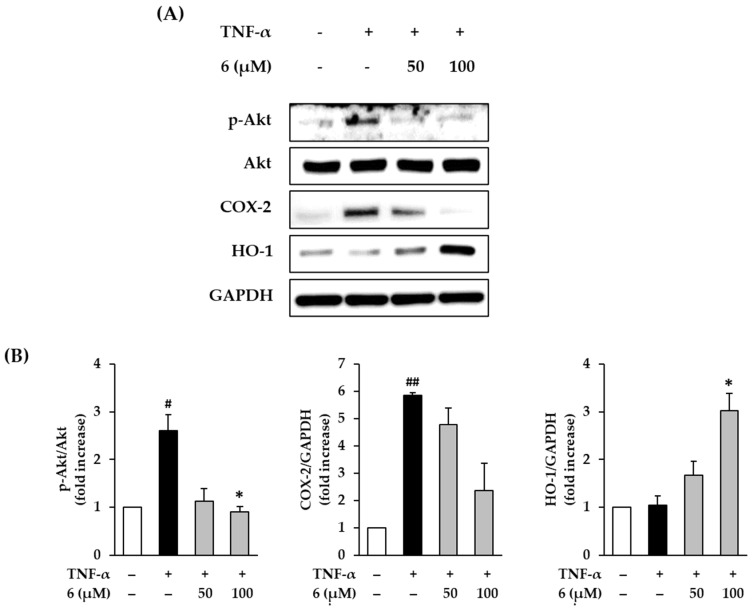
Effects of 3,5,7-trimethoxyflavone (6) on MAPK phosphorylation in TNF-α stimulated normal human dermal fibroblasts (NHDFs). (**A**) Bands of protein expressions. (**B**) Bar graphs of the relative expression. We seeded NHDFs at 3 × 10^5^ cells/well in 6-well plates, incubated for 24 h, and then replaced the medium with a serum-free medium to create starvation conditions. After 24 h, cells were treated with 50 and 100 μM of 6 for 1 h, and the cells were exposed to 20 ng/mL TNF-α for 6 h. Protein expressions of p-Akt, Akt, COX-2, HO-1, and GAPDH were determined with Western blotting. Results of each protein expression was presented as percent of the vehicle control. The results were obtained through three replicate experiments, and the graphs are represented as mean ± SEM. ^#^ *p* < 0.05 and ^##^ *p* < 0.01 vs. vehicle control. * *p* < 0.05 vs. TNF-α stimulated control.

Immoderate production of ROS activates Akt, which causes inflammation by upregulating COX-2 [46]. To determine the protective effect of 3,5,7-trimethoxyflavone (6) against the inflammatory response, we evaluated its effect on Akt phosphorylation and COX-2 expression in TNF-α-stimulated NHDFs. Western blotting was used to measure protein expression levels of p-Akt, Akt, and COX-2. As shown in Figure 6, 20 ng/mL TNF-α exposure induced Akt phosphorylation in NHDFs compared to that with the vehicle control, which was reduced upon treatment with 6. TNF-α stimulation increased Akt phosphorylation by 2.61 ± 0.38-fold, whereas treatment with 6 suppressed it by 1.13 ± 0.28-fold (50 µM) and 0.90 ± 0.12-fold (100 µM, *p* < 0.05) compared with that in the control. Further, TNF-α stimulation increased COX-2 expression by 5.85 ± 0.11-fold, whereas treatment with 6 suppressed it by 4.78 ± 0.61-fold (50 µM) and 2.36 ± 1.00-fold (100 µM) compared with that in the control; however, this difference was not significant. These results indicated that 3,5,7-trimethoxyflavone (6) suppressed the activation of inflammation by TNF-α stimulation. Therefore, 3,5,7-trimethoxyflavone has an ameliorating effect on the inflammation induced by ROS accumulation.

An increase in HO-1 through the transcriptional activation of Nrf2 suppresses free radical production, thereby preventing inflammatory damage and apoptosis in human skin cells [47,48]. In Figure 6, exposure of 20 ng/mL TNF-α did not show alteration of HO-1 expression in NHDFs compared to that in the vehicle control; however, this was increased by treatment with **6**. HO-1 expression increased by 1.68 ± 0.29-fold (50 µM) and 3.03 ± 0.36-fold (100 µM, *p* < 0.05) of the control upon treatment with **6**. These results suggest that 3,5,7-trimethoxyflavone (6) inhibits the ROS accumulation induced by TNF-α stimulation through free radical trapping by HO-1.

### 3.7. Effect of 3,5,7-Trimethoxyflavone on MMP-1 and Pro-Collagen I α1 mRNA and Protein Expression in TNF-α-Stimulated NHDFs

**Figure 7:**
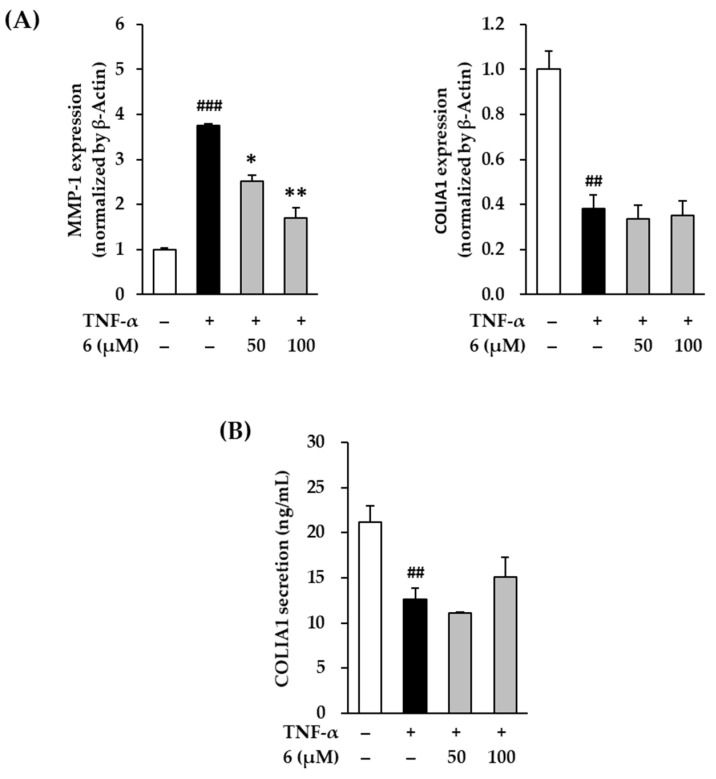
Effects of 3,5,7-trimethoxyflavone (6) on MMP-1 and COLIA1 in TNF-α stimulated normal human dermal fibroblasts (NHDFs). (**A**) The mRNA expressions of MMP-1 and COLIA1. We seeded NHDFs at 3 × 10^5^ cells/well in 6-well plates, incubated them for 24 h, and then replaced the medium with a serum-free medium to create starvation conditions. After 24 h, cells were treated with 50 and 100 μM of 6 for 1 h, and the cells were exposed to 20 ng/mL TNF-α for 12 h. The mRNA expressions of MMP-1 and COLIA1 were determined with RT-qPCR. Results of each mRNA expression was presented as percent of the vehicle control. (**B**) The protein secretion of COLIA1. We seeded NHDFs at or 2 × 10^4^ cells/well in 48-well plates, incubated them for 24 h, and then replaced the medium with a serum-free medium to create starvation conditions. After 24 h, NHDFs were exposed to 20 ng/mL TNF-α in the presence or absence of 6 for 24 h. The protein secretion of COLIA1 were determined with an ELISA kit. The results were obtained through three replicate experiments, and the graphs are represented as mean ± SEM. ^##^ *p* < 0.01 and ^###^ *p* < 0.001 vs. vehicle control. * *p* < 0.05 and ** *p* < 0.01 vs. TNF-α stimulated control.

UV radiation induces ROS accumulation, which alters the structure and function of genes and proteins, ultimately causing skin damage. It increases the expression and secretion of MMP-1 collagenase and induces collagen degradation. Eventually, the skin ECM disintegrates, leading to skin damage such as wrinkles [49,50]. Therefore, substances that inhibit collagenase activity are considered as potential therapeutic candidates for skin damage [51]. In Figure 7A, exposure of 20 ng/mL TNF-α did show increased MMP-1 expression in NHDFs compared to that in the vehicle control; this was reduced by 3,5,7-trimethoxyflavone (**6**) treatment. TNF-α stimulation also increased MMP-1 mRNA expression by 3.76 ± 0.03-fold, whereas treatment with **6** suppressed it by 2.53 ± 0.13-fold (50 µM) and 1.70 ± 0.23-fold (100 µM, *p* < 0.05) compared with that in the control. As shown in Figure 3, MMP-1 secretion was induced by TNF-α-stimulation, whereas treatment with 6 suppressed it. These results indicate that 3,5,7-trimethoxyflavone (**6**) inhibits MMP-1 expression induced by TNF-α stimulation. 

Procollagen is secreted out of the cell, a part of the N-terminal peptide is cleaved by enzymes, and the cleaved procollagen is combined with the help of oxygen, iron ions, and ascorbic acid to form collagen in the form of fibers [52]. Therefore, the expression of procollagen COLIA1 was examined to indirectly evaluate collagen synthesis. In the Figure 7A, exposure of 20 ng/mL TNF-α did show a decrease in COLIA1 expression in NHDFs compared to that in the vehicle control. TNF-α stimulation decreased COL1A1 mRNA expression to 0.38 ± 0.06-fold, but this remained unchanged upon treatment with 3,5,7-trimethoxyflavone (**6**) at 50 and 100 µM. In Figure 7B, COLIA1 secretion induced by TNF-α stimulation decreased to 12.59 ± 1.24 ng/mL, compared with that in the vehicle control (21.13 ± 1.86 ng/mL). Treatment with 100 µM of **6** increased COLIA1 to 15.65 ± 0.46 ng/mL; however, this difference was not significant. Despite the lack of significance, this result indicated that 3,5,7-trimethoxyflavone (**6**) can reverse the decrease in COLIA1. In summary, these results suggest that 3,5,7-trimethoxyflavone (**6**) has the potential to suppress the advanced cutaneous ECM degradation induced by oxidative stress.

### 3.8. Effect of 3,5,7-Trimethoxyflavone on Proinflammatory Cytokines in TNF-α-Stimulated NHDFs

When oxidative stress occurs, the response is hypersecretion of pro-inflammatory cytokines, including TNF-α, IL-1β, IL-6, and IL-8 [53]. Consequently, the inflammatory response is upregulated, leading to skin aging and various lesions [6,54]. To evaluate the effect of 3,5,7-trimethoxyflavone (**6**) on the inflammation-related responses in skin cells, IL-1β, IL-6, and IL-8 levels were evaluated in TNF-α-stimulated NHDFs.

**Figure 8:**
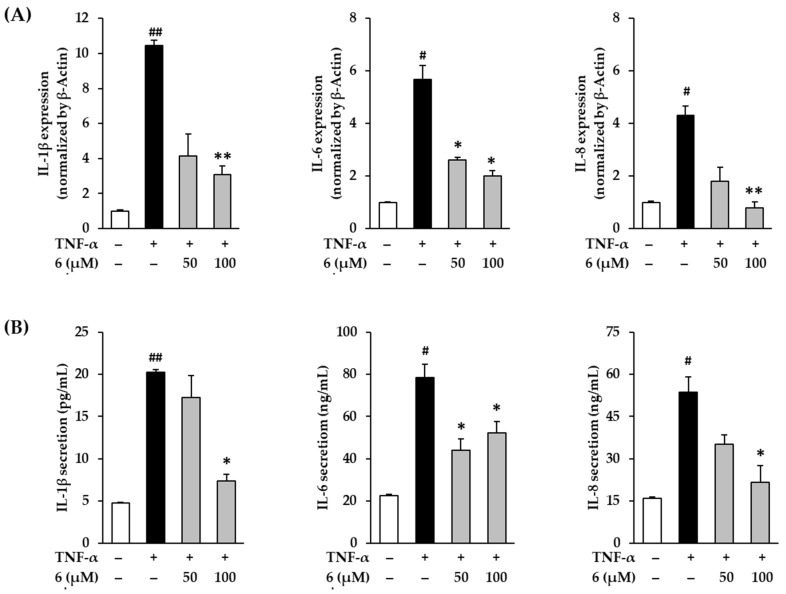
Effects of 3,5,7-trimethoxyflavone (6) on proinflammatory cytokines such as IL-1β, IL-6, and IL-8 in normal human dermal fibroblasts (NHDFs) stimulated by TNF-α. (**A**) The mRNA expressions of IL-1β, IL-6, and IL-8. We seeded NHDFs at 3 × 10^5^ cells/well in 6-well plates, incubated them for 24 h, and then replaced the medium with a serum-free medium to create starvation conditions. After 24 h, NHDFs were exposed to 20 ng/mL TNF-α in the presence or absence of 6 for 4 h. The mRNA expressions of IL-1β, IL-6, and IL-8 were determined with RT-qPCR. Results of each mRNA expression was presented as percent of the vehicle control. (**B**) The protein secretion of IL-1β, IL-6, and IL-8. We seeded NHDFs at or 2 × 10^4^ cells/well in 48-well plates, and incubated them for 24 h, and then replaced the medium with a serum-free medium to create starvation conditions. After 24 h, cells were treated with 3,5,7-trimethoxyflavone for 24 h. The cells were then exposed to 20 ng/mL TNF-α for 12 h. The protein secretion of IL-1β, IL-6, and IL-8 were determined with an ELISA kit. The experiments were performed in triplicate, and the graphs are represented as mean ± standard error of the mean (SEM). ^#^ *p* < 0.05 and ^##^ *p* < 0.01 vs. vehicle control. * *p* < 0.05 and ** *p* < 0.01 vs. TNF-α stimulated control.

In Figure 8A, exposure of 20 ng/mL TNF-α did show an increase in IL-1β, IL-6, and IL-8 in NHDFs compared to the vehicle control, which was reduced by 3,5,7-trimethoxyflavone (**6**) treatment. IL-1β mRNA expression increased by 10.45 ± 0.29-fold after TNF-α-stimulation (*p* < 0.01); however, treatment with **6** suppressed it by 4.16 ± 1.24-fold (50 µM, *p* < 0.01) and 3.08 ± 0.53-fold (100 µM, *p* < 0.01) of the control. IL-6 mRNA expression increased by 5.68 ± 0.53-fold (*p* < 0.05) after TNF-α stimulation; however, treatment with **6** suppressed it by 2.60 ± 0.10-fold (50 µM, *p* < 0.05) and 2.01 ± 0.20-fold (100 µM, *p* < 0.05) of the control. IL-8 mRNA expression increased by 6.24 ± 0.30-fold (*p* < 0.05) TNF-α stimulation; however, treatment with **6** suppressed it by 3.83 ± 0.92-fold (50 µM) and 2.38 ± 0.38-fold (100 µM, *p* < 0.01) of the control. As shown in Figure 8B, the secretion of IL-1β increased to 20.30 ± 0.27 pg/mL (*p* < 0.01) after TNF-α-stimulation compared with vehicle control (4.77 ± 0.04 pg/mL), and treatment with **6** suppressed it to 17.25 ± 2.61 ng/mL (50 µM) and 7.34 ± 0.84 pg/mL (100 µM, *p* < 0.05). The secretion of IL-6 increased to 78.51 ± 6.32 ng/mL (*p* < 0.05) after TNF-α-stimulation compared with vehicle control (22.51 ± 0.63 ng/mL), and treatment with **6** suppressed it to 43.98 ± 5.47 ng/mL (50 µM, *p* < 0.05) and 53.29 ± 5.37 ng/mL (100 µM, *p* < 0.05). The secretion of IL-8 increased to 53.77 ± 5.37 ng/mL (*p* < 0.05) after TNF-α-stimulation compared with vehicle control (15.87 ± 0.53 ng/mL), and treatment with **6** suppressed it to 35.14 ± 3.37 ng/mL (50 µM) and 21.66 ± 5.89 ng/mL (100 µM, *p* < 0.05). 

These results indicate that 3,5,7-trimethoxyflavone (6) suppresses TNF-α stimulation-induced proinflammatory cytokines IL-1β, IL-6, and IL-8. Therefore, 3,5,7-trimethoxyflavone (6) has the potential to suppress inflammatory responses in the skin caused by proinflammatory cytokines. 

## 4. Discussion

The skin is the largest organ of the body. It acts as a barrier against chemical and physical pollutants and protects internal organs. Proteins of extracellular matrix are generated by fibroblasts present in the dermis and are accountable for the elasticity and strength of the skin. Breakdown of ECM from the dermis results in skin aging. There are many types of collagens in the dermal ECM (III, V, VII); however, type I collagen is the most important structural protein. The main cause of aged skin that appears thin, smooth, dry, and inelastic is a decrease in collagen fibers and type I collagen production. In the process of skin aging, the collagenase MMP-1 destroys collagen fibrils. It has been shown that with aging, MMP-1 levels increase and collagen expression in human skin decreases. Furthermore, excessive ECM degradation of the dermis by MMP-1 contributes to inflammation of connective tissue. Therefore, the balance between MMP 1 and type I collagen expression plays an important role in skin aging [55].

The skin can age internally by a temporal aging process that affects all body organs or externally because of environmental factors, such as sun exposure and smoking. External and internal skin aging produces inflammatory cytokines, which are closely related to the skin inflammation caused by them. In the process of intrinsic aging and extrinsic aging, various cells of the skin produce the inflammatory cytokine TNF-α, which functions as a major regulator of cell metabolism and activity. UV irradiation stimulates cell surface receptors, which are the main contributors of skin aging. Of the ligands for these receptors, TNF-α induces increased MMP expression and decreased collagen expression, leading to ECM disruption. Moreover, TNF-α is considered an important modulator of inflammatory dermatological symptoms and diseases. TNF-α increases pro-inflammatory cytokines such as IL-1 and IL-6, which activate NF-κB [25,56,57]. Therefore, regulation of TNF-α activity can be an attractive strategic method for searching for candidate substances to prevent skin damage and aging [55,58].

In the present research, we studied the effects of methoxyflavones isolated from black ginger (*K. parviflora*), including 5-hydroxy-7-methoxyflavone (1), 3,7-dimethoxy-5-hydroxyflavone (2), 5-hydroxy-3,7,3,4-tetramethoxyflavone (3), 7,4-dimethylapigenin (4), 3,7,4-trimethylkaempferol (5), and 3,5,7-trimethoxyflavone (6) on TNF-α-induced damage to normal human dermal fibroblasts (NHDF) with the measurement of MMP-1 and COLIA1 expression. Among the methoxyflavones, 3,5,7-trimethoxyflavone (6) had the intensive ability to protect fibroblasts that were damaged by TNF-α, which inhibited MMP-1 secretion. Based on the activities observed, structure-activity relationship (SAR) can be investigated through four groups of the isolated compounds, including (1) compounds **1** and **4**, (2) compounds **2** and **6**, (3) compounds **3** and **5**, and (4) compounds **4** and **5**. The SAR analysis suggested that the 3-OCH_3_ and methoxyl groups in B-ring are not associated with the protective effects based on the activities of 1/4, 3/5, and 4/5. In contrast, the 5-OCH_3_ moiety played a critical role in the activity, considering that compound **6** with 5-OCH_3_ showed the potent activity, whereas compound 2 with 5-OH was inactive. 

COX-2, IL-1, and IL-6 are pro-inflammatory mediators that play a major role in causing skin aging and inflammatory skin symptoms. In skin aging, COX-2 acts as a mediator for the biosynthesis of prostaglandin E2, which contributes to the production of MMPs. IL-1 and IL-6 are regulators that contribute to skin wrinkle formation, including inhibition of collagen biosynthesis [59]. Luteolin, a flavonoid isolated and reported from various natural products, suppresses ROS and pro-inflammatory mediators and promotes photoaging of the epidermis and dermis to reduce IL-6, IL-22, IL-17, and COX-2 [46]. Similarly, 3,5,7-trimethoxyflavone (6) significantly inhibits TNF-α-induced expression of IL-1β, IL-6, and COX-2 and is expected to have a protective effect on skin damage based on inhibition of inflammation-related reactions.

Oxidative stress is also an important factor related to skin aging. Reactive oxygen species generated by intrinsic (chronic aging) and total permeable aging (optical) stimulate MMP expression, suppress growth factor-beta signaling, suppress collagen degradation, and trigger collagen fibrous biosynthesis [60]. Therefore, development of skin antioxidants with natural products is one of the major strategies of skincare product development in the cosmetics industry [61]. According to a recent study by Li et al., orange low-temperature crimping oil has a high content of polymethoxyflavones and was effective in preventing UVB-induced oxidative damage in mouse skin [62]. Similarly, in this study, 3,5,7-trimethoxyflavone (6) had the effect of suppressing the reactive oxygen species produced by stimulation with TNF-α in NHDFs. The position and number of methoxy groups in the polymethoxyflavone structure related to the antioxidant properties, suggest a relevance for these pharmacological effects.

MAPK plays a central role in controlling skin damage in combination with two downstream pathways associated with oxidative stress and inflammatory responses, AP-1 and NF-κB [62,63]. In addition, activation of MAPK induces phosphorylation and rearrangement of NF-κB, producing MMP-1 and inflammatory cytokines [62,64]. Therefore, retrograde movement of this pathway plays an important role in delaying skin aging. Previous reports have shown that treatment with MAPK inhibitors reversed the photoaging process [19,64]. NHDFs treated with 3,5,7-trimethoxyflavone (6) showed diminished phosphorylation of MAPK. This resulted in inhibition of MMP-1 production and collagen degradation. In this study, 3,5,7-trimethoxyflavone (6) has also been shown as a potential MAPK, Akt, and COX-2 inhibitor. NHDFs treated with 3,5,7-trimethoxyflavone (6) showed reduced phosphorylation of MAPK, reduced phosphorylation of Akt, and reduced COX-2.

In summary, among the methoxyflavones isolated from black ginger (*K. parviflora*), 3,5,7-trimethoxyflavone (6) has potent preventive effect in TNF-α-induced injury to HDFs by suppressing MMP-1 expression and recovery COLIA1 expression. In TNF-α-stimulated HDF, the skin protection mechanism of 3,5,7-trimethoxyflavone (6) involves the reduction of reactive oxygen species and anti-inflammatory mediators, including COX-2, IL-1, IL-6, and IL-8 production, through the inhibition of oxidative stress and inflammatory response. Moreover, 3,5,7-trimethoxyflavone (6) alleviates the phosphorylation of MAPK and Akt, which inhibits MMP-1 production and collagen fiber degradation.

However, this study had some limitations. For example, the potential of 3,5,7-trimethoxyflavone (6) to prevent skin aging has been studied only in HDFs. To completely understand the anti-aging effect of 3,5,7-trimethoxyflavone (6), research needs to be extended to many different cell lines such as melanocytes, keratinocytes, and organic 3D skin models. 

Our previous study has shown that 5,7,4′trimethoxyflavone is effective at low concentrations of 6.25 and 12.5 μM [63]. On Because 3,5,7-trimethoxyflavone (6) compound 6 is effective at 50 or more, the question of weak activity may be raised. However, skin improvers directly penetrate the skin to show their effects. Therefore, the active compound must penetrate the skin barrier without causing skin irritation. Recent studies have reported that the microemulsion formulation quercetin increases skin penetration [64]. Therefore, it is believed that additional studies such as topical and transdermal delivery are needed to determine if a physiological approach is possible.

## 5. Conclusions

The results of this study indicate that 3,5,7-trimethoxyflavone (**6**) isolated from *K. parviflora* has inhibitory effects on TNF-α-induced MMP-1 in NHDFs. It was found that 3,5,7-trimethoxyflavone inhibited TNF-α-induced ROS, which plays a key role in the inflammatory response and ECM degradation that occurs during skin damage, such as skin aging and various cutaneous lesions. The changes by which 3,5,7-trimethoxyflavone (**6**) reduces TNF-α-stimulated responses in NHDFs are correlated with the suppression of Akt, COX-2, MAPK activation, and induction of HO-1. Moreover, 3,5,7-trimethoxyflavone reduced the expression of proinflammatory cytokine mediators induced by TNF-α stimulation, including IL-1β, IL-6, and IL-8. These findings provide evidence that 3,5,7-trimethoxyflavones may protect against skin damage caused by accumulation of oxidative stress. Additional experiments are required to fully understand the mechanism of 3,5,7-trimethoxyflavone activity; however, it is a potential substance for preventing skin damage, including skin aging and various skin lesions. 

## Data Availability

Data is contained within the article and supplementary materials.

## Supplementary Materials

The following are available online at https://www.mdpi.com/article/10.3390/antiox11020425/s1, Figure S1: ^1^H-NMR spectrum of compound **1** (in CDCl_3_), Figure S2: ^1^H-NMR spectrum of compound **2** (in CDCl_3_), Figure S3: ^1^H-NMR spectrum of compound **3** (in CDCl_3_), Figure S4: ^1^H-NMR spectrum of compound **4** (in CDCl_3_), Figure S5: ^1^H-NMR spectrum of compound **5** (in CDCl_3_), Figure S6: ^1^H-NMR spectrum of compound **6** (in CDCl_3_).
