# Supplementary material for: Improvement of Damage in Human Dermal Fibroblasts by 3,5,7-Trimethoxyflavone from Black Ginger (Kaempferia parviflora)
Source: Antioxidants (Basel). 2022 Feb 19;11(2):425. doi: 10.3390/antiox11020425 (PMC8869600; doi:10.3390/antiox11020425)
Supplement: Supplementary file 1 [file antioxidants-11-00425-s001.zip › antioxidants-1541957-supplementary.pdf]

Supplementary Materials

Improvement of Damage in Human Dermal Fibroblasts by 3,5,7-Trimethoxyflavone from Black Ginger (*Kaempferia parviflora*)

Sullim Lee[1], Taesu Jang[2], Ki Hyun Kim[3,*] and Ki Sung Kang [4,*]

[1] Department of Life Science, College of Bio-Nano Technology, Gachon University, Seongnam 13120, Korea; sullimlee@gachon.ac.kr (S.L.)
[2] College of Medicine, Dankook University, Cheonan 31116, Korea; jangts@dankook.ac.kr (T.J.)
[3] School of Pharmacy, Sungkyunkwan University, Suwon 16419, Korea; khkim83@skku.edu (K.H.K)
[4] College of Korean Medicine, Gachon University, Seongnam 13120, Korea; kkang@gachon.ac.kr (K.S.K.)

* Correspondence: Tel.: 82-31-290-7700; E-mail: khkim83@skku.edu (K.H.K); Tel.: 82-31-750-5402; Fax: 82-31-750-5416; E-mail: kkang@gachon.ac.kr (K.S.K.)

Figure S1 : ^{1}H-NMR spectrum of compound **1** (in $CDCl_3$)
Figure S2 : ^{1}H-NMR spectrum of compound **2** (in $CDCl_3$)
Figure S3 : ^{1}H-NMR spectrum of compound **3** (in $CDCl_3$)
Figure S4 : ^{1}H-NMR spectrum of compound **4** (in $CDCl_3$)
Figure S5 : ^{1}H-NMR spectrum of compound **5** (in $CDCl_3$)
Figure S6 : ^{1}H-NMR spectrum of compound **6** (in $CDCl_3$)

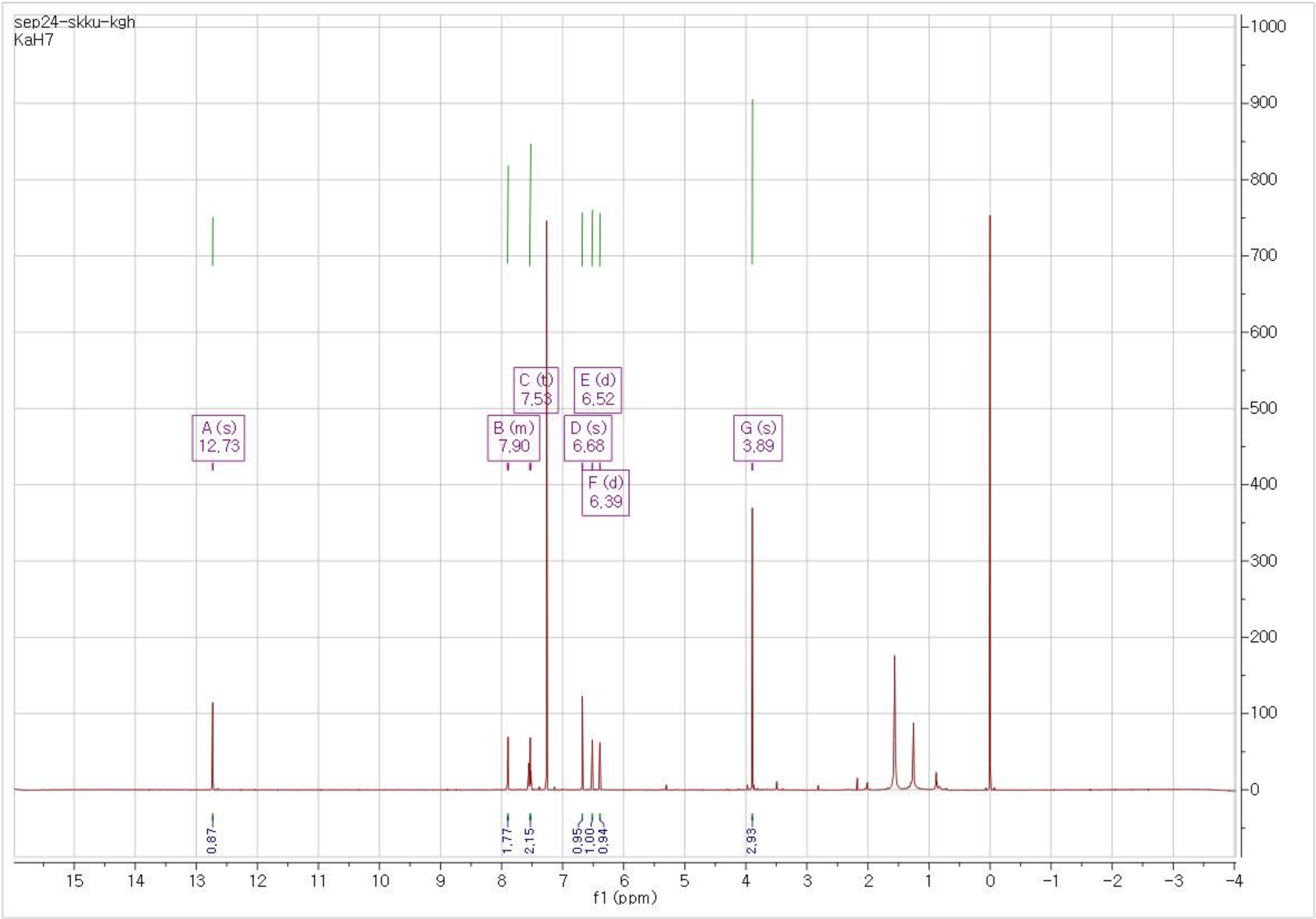

Figure S1 : ^{1}H-NMR spectrum of compound **1** (in $CDCl_3$)

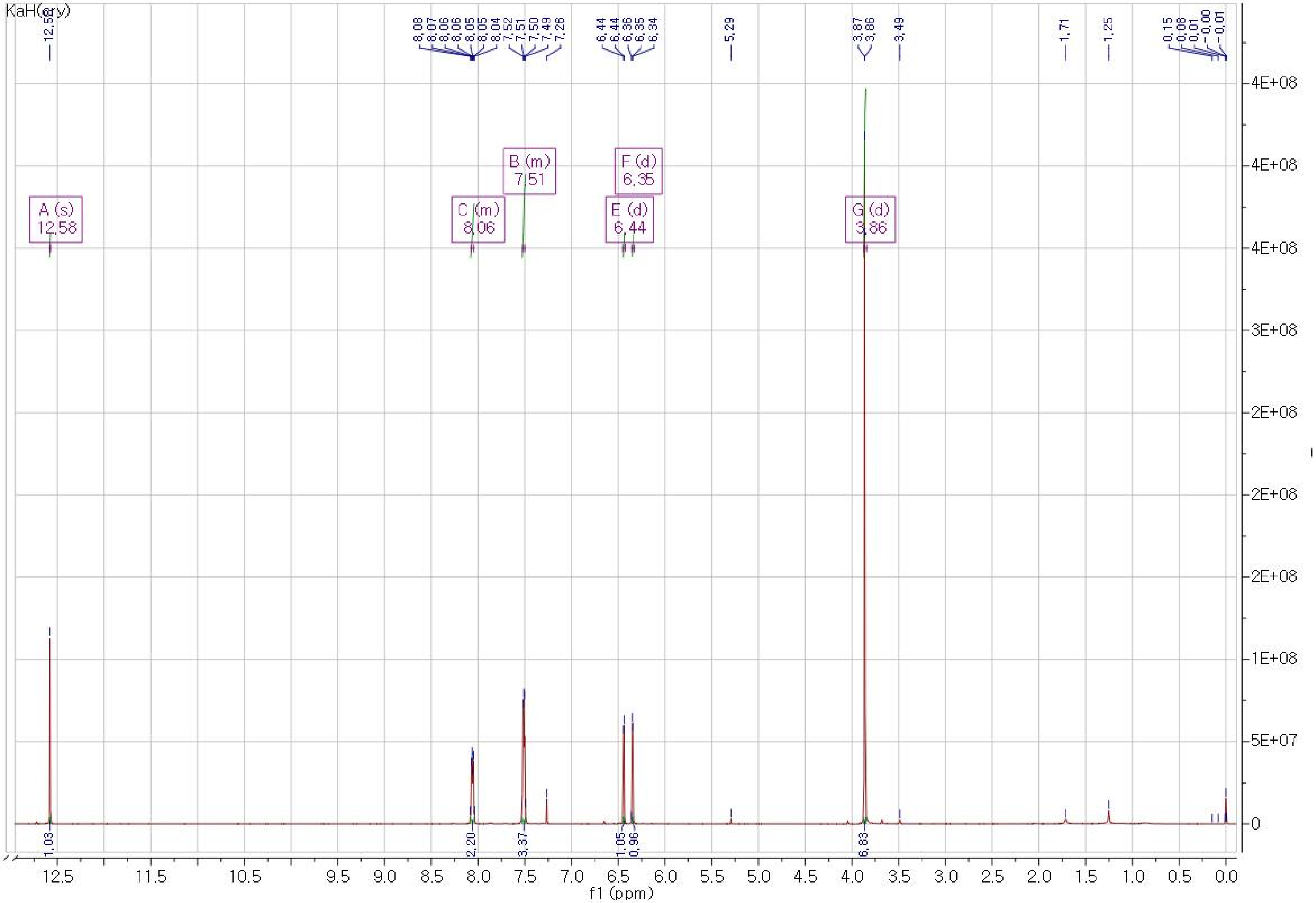

Figure S2 : ^{1}H-NMR spectrum of compound **2** (in $CDCl_3$)

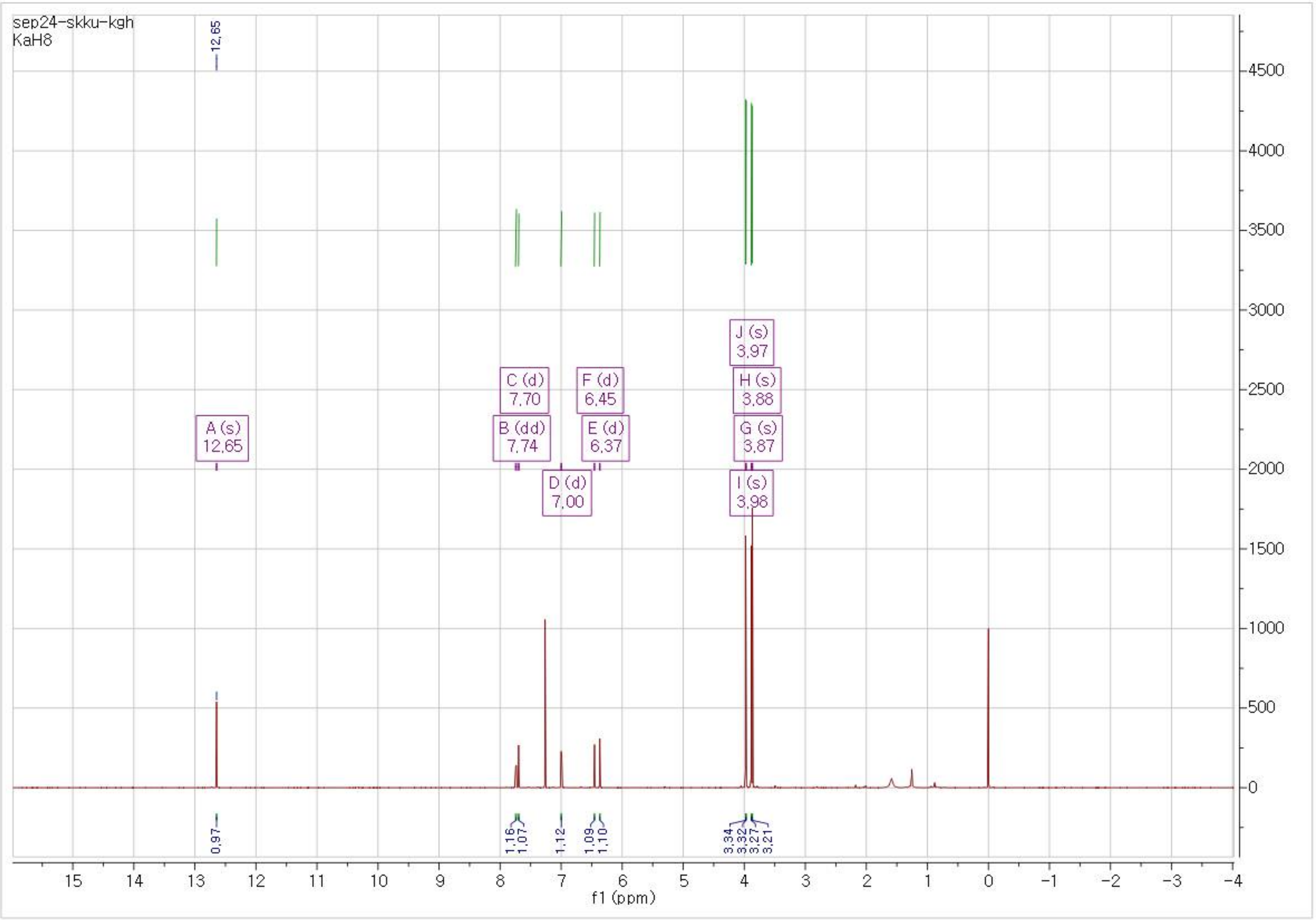

Figure S3 : ^{1}H-NMR spectrum of compound **3** (in $CDCl_3$)

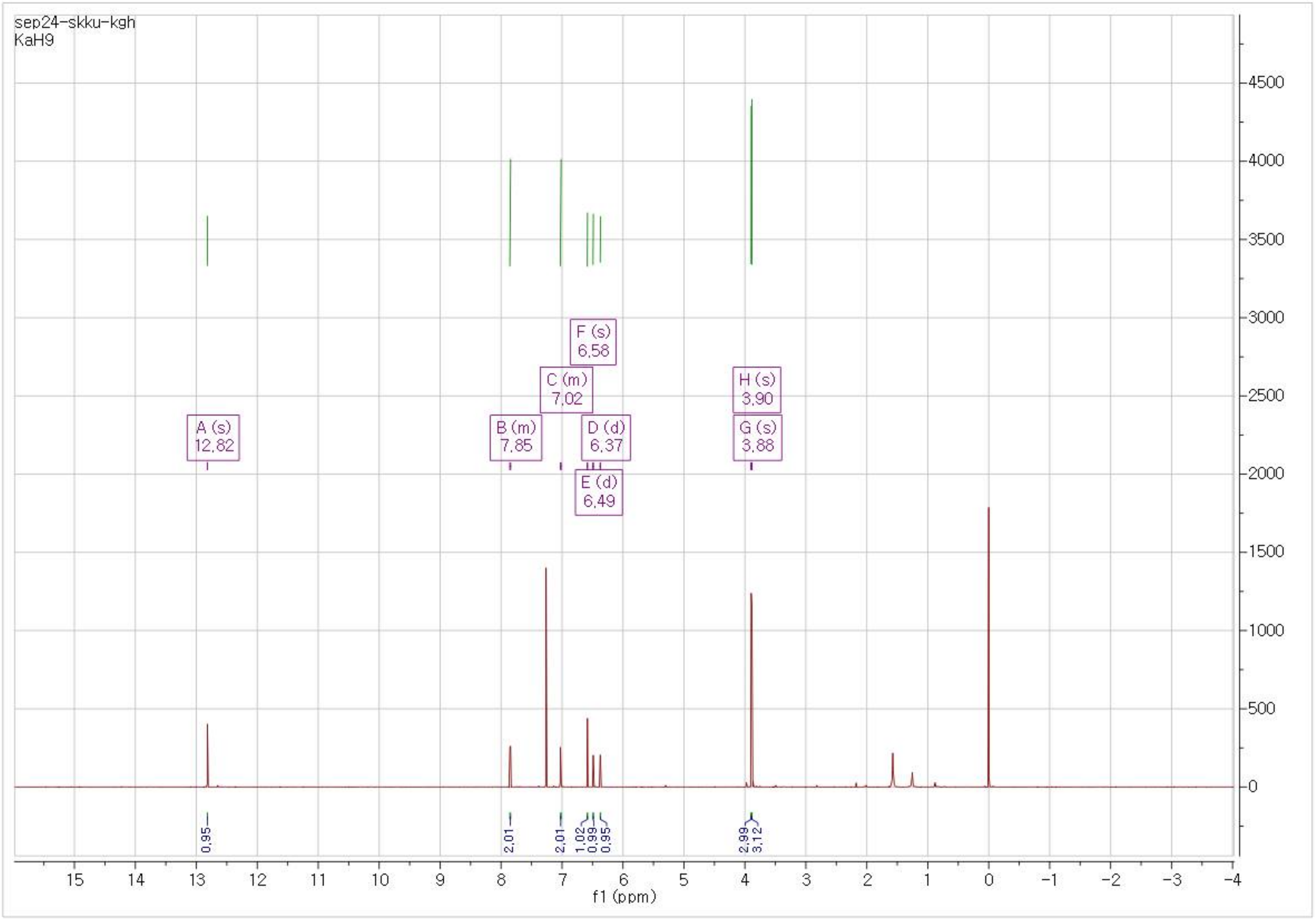

Figure S4 : ¹H-NMR spectrum of compound **4** (in $CDCl_3$)

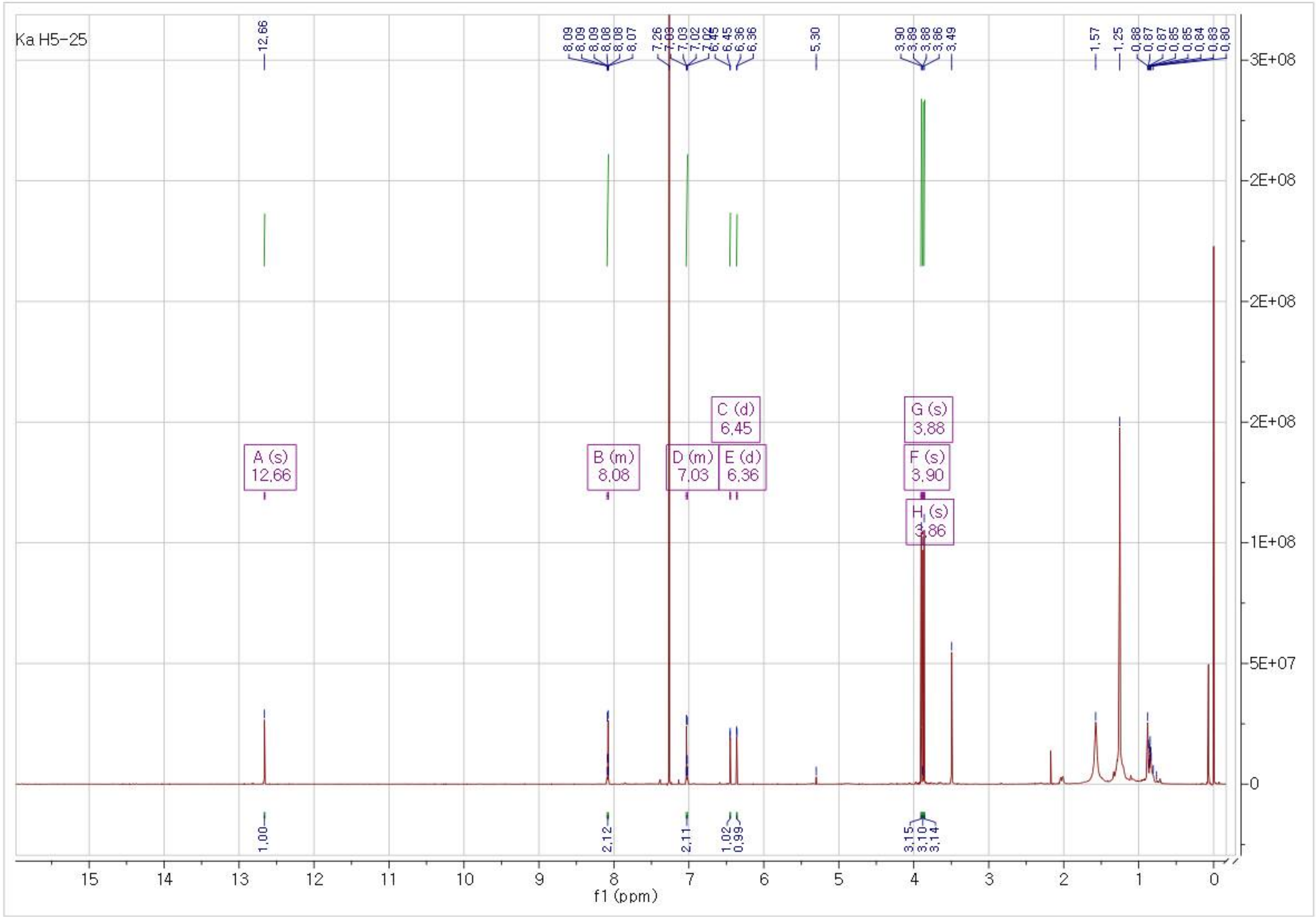

Figure S5 : ^{1}H-NMR spectrum of compound **5** (in $CDCl_3$)

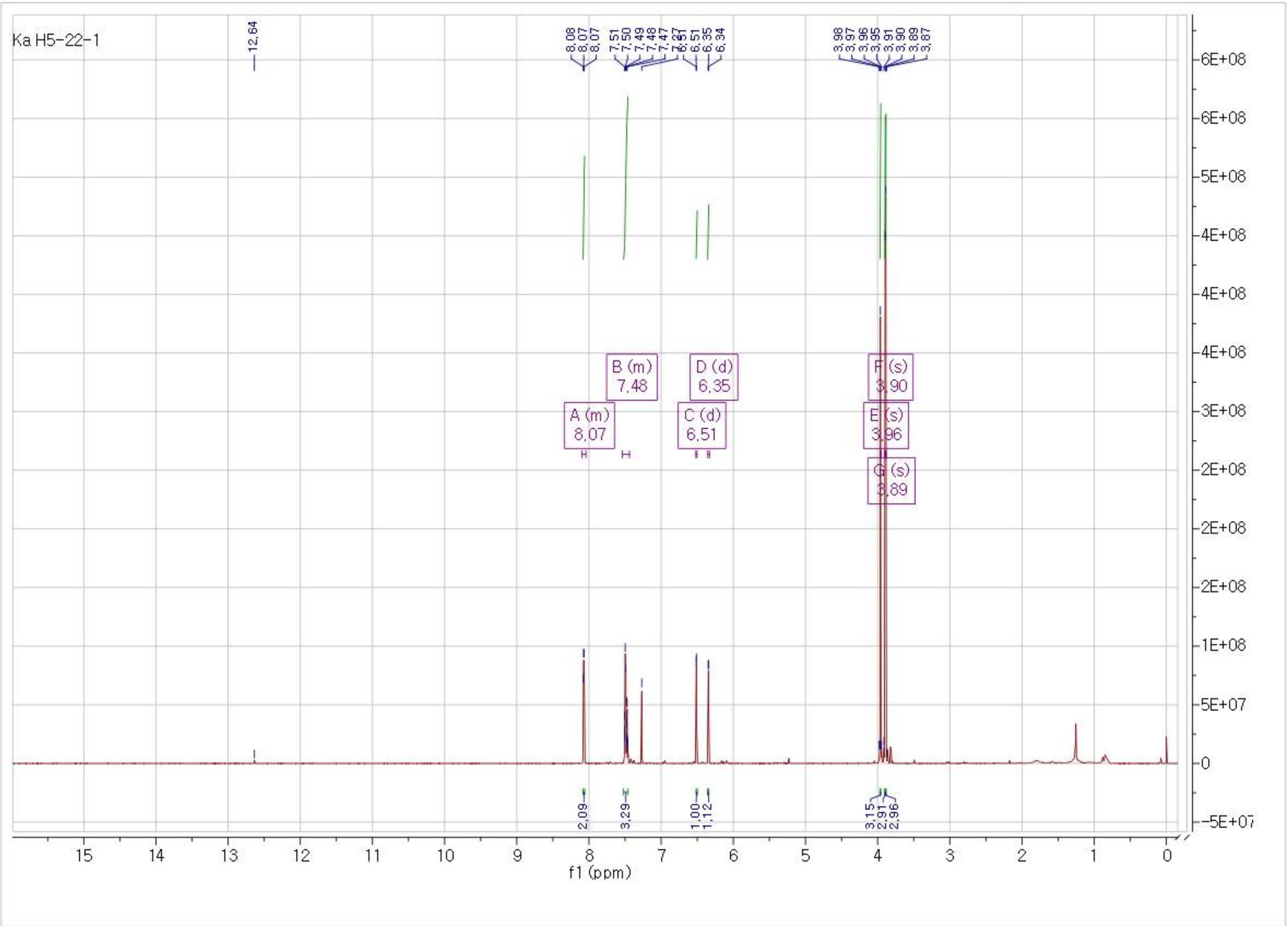

Figure S6 : ^{1}H-NMR spectrum of compound **6** (in $CDCl_3$)
